# Airway remodeling in horses with mild and moderate asthma

**DOI:** 10.1111/jvim.16333

**Published:** 2021-12-08

**Authors:** Amandine Bessonnat, Pierre Hélie, Carolyn Grimes, Jean‐Pierre Lavoie

**Affiliations:** ^1^ Department of Clinical Sciences Faculty of Veterinary Medicine, University of Montreal Saint‐Hyacinthe Quebec Canada; ^2^ Faculty of Veterinary Medicine, Department of Pathology and Microbiology University of Montreal Saint‐Hyacinthe Quebec Canada; ^3^ Present address: Zoetis Reference Laboratories San Diego California USA

**Keywords:** central airway, endobronchial biopsy, histomorphometry, semiquantitative histologic score

## Abstract

**Background:**

There is a remodeling of the central airways in horses with severe asthma but whether a similar process occurs in horses with the mild or moderate asthma (MMA) is unknown.

**Objectives:**

To evaluate lesions affecting the central airways of horses with MMA.

**Animals:**

Twelve horses with MMA and 8 control horses.

**Methods:**

Case‐control retrospective study of horses classified as MMA affected or controls based on history and bronchoalveolar lavage fluid cytology. Endobronchial biopsies were analyzed using histomorphometry and a semiquantitative histologic scoring system.

**Results:**

Histomorphometry identified epithelial hyperplasia (47 μm^2^/μm [34‐57 μm^2^/μm]; *P* = .02), a thickened lamina propria (166 μm [73‐336 μm]; *P* = .04), and smooth muscle fibrosis (42% [33%‐78%]; *P* = .04) in horses with MMA when compared to controls horses (24 μm^2^/μm [21‐80 μm^2^/μm]; 76 μm [36‐176 μm]; and 33% [26%‐52%], respectively). The semiquantitative score results indicated, in horses with MMA, the presence of epithelial hyperplasia (7 of the 12 horses with MMA and only 1 of the 8 control horses had a score of 1/1), and submucosal inflammatory leucocytes in the central airway (11 of the 12 horses with MMA and only 4 of the 8 control horses had a score ≥ 1/2).

**Conclusions and Clinical Relevance:**

Tissue remodeling of the bronchial lamina propria, epithelium, and smooth muscle was present in horses with MMA.

**Abbreviations**
ASM: airway smooth muscle
BAL: bronchoalveolar lavage
HEPS: hematoxylin‐eosin‐phloxine‐saffron
MMA: mild or moderate equine asthma
SA: severe asthma

## 1 INTRODUCTION

Mild or moderate asthma (MMA; also known as inflammatory airway disease) in horses is associated with lower airway obstruction^1^, ^2^ and is a common cause of exercise intolerance in performance horses of all ages.^3^, ^4^ Eighty percent of racehorses in their first year of training experience lower airway inflammation^5^ and airway inflammation is associated with decreased racing speed.^6^ The condition could be underdiagnosed because affected horses often do not have clinical signs suggestive of a lower airway disease.^7^

In clinical practice, the diagnosis of MMA is based on the presence of compatible clinical signs combined with abnormal bronchoalveolar lavage (BAL) fluid cytology.^8^ Laboratory studies reveal abnormal lung function at rest,^9^ decreased PaO_2_,^10^ and decreased VO_2_ peak during exercise.^11^ Although diagnostic tools are helpful to study the physiological effects of MMA, use is currently limited by the specialized equipment and expertise required. Bronchoalveolar lavage, however, can easily be performed in the field.

Remodeling in the central and peripheral airways of horses with severe asthma (SA) affect the epithelium, the lamina propria, and the bronchial smooth muscle layer.^12^, ^13^, ^14^, ^15^, ^16^, ^17^, ^18^, ^19^, ^20^ These structural changes correlate with decreased lung function in horses with SA, and could contribute to the impaired gas exchanged during exercise in MMA.^17^, ^20^, ^21^, ^22^, ^23^ Airway inflammation and obstruction are usually managed therapeutically and airway remodeling in SA is only partially reversed by antigen avoidance, and administration of corticosteroids and bronchodilators.^21^, ^24^ Although there is limited information about airway remodeling in horses with MMA, the presence of histological changes might be associated with the severity of the clinical signs.^20^, ^25^, ^26^

Considering the presence of lower airway obstruction in horses with asthma of all severities, we postulated that lesions are present in central airways of horses with MMA. Our objective was to assess the histologic alterations in the central airways of horses with MMA using histomorphometry and a semiquantitative scoring system.

## 2 MATERIAL AND METHODS

History, CBC, serum biochemistry, BAL cytology differential count, and endobronchial biopsies from horses with MMA and control horses from the Equine Respiratory Tissue Bank were studied. Characteristics of horses with MMA were previously reported.^27^ This exploratory study was retrospective, controlled, and blinded. The experimental protocol was approved by the University of Montreal's Animal Care Committee (Rech‐1647) and informed consent was provided by all owners.

### 2.1 Horses

Thirty‐four client‐owned horses were examined at the Equine Hospital of the Faculty of Veterinary Medicine between 2015 and 2016 (1 horse in 2014) for coughing at rest or during exercise, exercise intolerance, or breathing difficulties. Horses with MMA were included if BAL cytology was inflammatory with at least 1 of the following parameters: neutrophils ≥5%; mast cells ≥2%; or eosinophils ≥1%. Twenty‐four control horses that were client‐owned or from the research/teaching herd were examined between 2015 and 2016 (1 horse in 2013), had no history of clinical signs of airway disease. Control horses were included if BAL cytology was normal. Horses from both groups had normal CBC and serum biochemistry results and were considered otherwise healthy based on a complete physical examination. Horses were excluded if BAL cytology or endobronchial biopsy, or both diagnostic tests were not available, if they had a history of labored breathing at rest (dyspnea) suggestive of SA, if BAL neutrophilia was severe (>25%),^28^ or if horses had a known concomitant disease.

### 2.2 BAL fluid collection and analysis

Collection of BAL fluid was performed as described.^28^ The BAL differential count was made on archived samples blindly and in accordance with previously described methods^27^ by a board‐certified clinical pathologist (C. Grimes).

### 2.3 Endobronchial biopsies

Endobronchial biopsies were obtained by videoendoscopy after collection of the BAL fluid. Briefly, the bronchoscope was advanced until it wedged to the most distal airway, and a smooth oval disposable biopsy forceps (FB‐234U, Olympus, Richmond Hill, Canada; diameter, 2.85 mm) was used to sample sequentially the most caudal to the most cranial carina until reaching the main carina.^13^ Samples were fixed in 10% neutral‐buffered formalin for 48 to 72 hours before paraffin embedding.^29^

### 2.4 Histopathologic analysis

Biopsies were stained with alcian blue and hematoxylin‐eosin‐phloxine‐saffron (HEPS) and with modified Russell‐Movat's pentachrome as previously described.^30^ Twenty‐four biopsies in horses with MMA (2 biopsies/horse) and 18 biopsies of control horses (from 2 to 4 biopsies/horse) reaching the inclusion criteria were stained by modified Russell‐Movat's pentachrome and were scored based on quality (1‐5/5).^13^ The quality score evaluates the orientation, architecture, structure preservation, and presence of airway smooth muscle (ASM) in the biopsies. A score of 1/5 represents a poor‐quality tissue where the continuity between epithelium, extracellular matrix (ECM), and smooth muscle is lost. A score of 2/5 represents a well‐oriented tissue, but its architecture is not completely preserved. A score of 3/5 represents a well‐oriented tissue, and its architecture is preserved at least in 50% of the biopsy. A score of 4/5 represents an optimal tissue orientation for the biopsy with of loss of continuity between tissues. Finally, a score of 5/5 represents an optimal tissue orientation for the biopsy, the tissue architecture is perfectly conserved, and the parenchymal borders of the smooth muscle layer are clearly identifiable. Biopsies with a score ≥ 3/5 were studied.

Twenty‐six biopsies from MMA and control horses were analyzed.

Twelve horses with MMA were included in the study (biopsies from 11 horses with Movat staining and 1 horse with HEPS staining). For the 11 horses with Movat staining, 14 biopsies with a quality score ≥ 3/5 were included (1 biopsy/horse; n = 8 and 2 biopsies/horse; n = 3). In addition, for 2 asthmatic horses 1 biopsy with a lower quality score (2/5; good tissue orientation, tissue architecture not completely preserved) but acceptable to perform measurement were also included.

Nine Movat‐stained biopsies with a quality score ≥ 3/5 (1 biopsy/horse; n = 7 and 2 biopsies/horse; n = 1) from 8 control horses were studied.

Histologic features assessed included: biopsy total area, smooth muscle (total and relative area), fibrosis within the smooth muscle, epithelium (total and relative area), goblet cells in the surface epithelium, airway extracellular matrix (total and relative area), and elastic fiber in the airway extracellular matrix: They were measured using point counting (200 points/tissue) in Movat stained biopsies using newCAST software (Visiopharm [version 6.5.2.2303], Hoersholm, Denmark).^31^ Basement membrane thickness, distance between basement membrane and muscle (evaluating lamina propria thickness), and epithelium area corrected by basement membrane length (evaluating epithelium hyperplasia) were measured on perpendicularly sliced Movat‐stained biopsies using ImageJ software (ImageJ [version 1.48v and 1.50h], National Institutes of Health, Bethesda, Maryland). When 2 biopsies per horse were available, the means of histomorphometric measurements for each criterion were used. The median value of measurements for each criterion was reported. Distances were measured in μm, surfaces areas in μm^2^, and ratios in percentage.

In 1 asthmatic horse, the histomorphometry analysis was performed on an HEPS stained biopsy; fibrosis within the smooth muscle, goblet cells in the surface epithelium, and elastic fibers in the airway extracellular matrix could not be evaluated.

Biopsies were evaluated by a board‐certified anatomic pathologist (P. Hélie) blinded to horse identity using a semiquantitative histologic score previously described for horses with SA.^14^ The pathologist attributed 0, 1, or 2 points in accordance with 10 predefined criteria. A 1‐scaled score (0 or 1/1) indicates the absence or the presence of the criterion (0 and 1 respectively), and a 2‐scaled score (0, 1 or 2/2) was used when the criterion was absent (0), moderate (1), or severe (2). Briefly, the epithelium was evaluated for the presence of hyperplasia (/1), inflammatory infiltrate (/2), goblet cell hyperplasia (/1), and desquamation (<10% of epithelium affected, 10%‐50% or >50%; /2). The extracellular matrix was evaluated for the thickness of the basement membrane (/1), submucosal inflammatory cells (/2), and mucous glands (/1). Finally, smooth muscle was evaluated for the presence of fibrosis (/2), mucous glands (/1), and smooth muscle ending visible (/1). The score was read on Movat‐stained biopsies for 18 horses (11 MMA and 7 control horses). For 1 horse with MMA, the score was read on a HEPS‐ and alcian blue‐stained biopsy. For 1 control horse, the score was read on a Movat‐stained biopsy completed with HEPS‐ and alcian blue‐stained biopsies. When 2 biopsies per horse were available, each parameter of the semiquantitative score was read on the tissue with the highest quality. Therefore, only 1 semiquantitative score (/14) per horse was obtained.

### 2.5 Statistical analysis

The Mann‐Whitney *U* test was used to evaluate difference between MMA and control groups regarding BAL cytology differential counts, histomorphometric measurements. A Spearman correlation was used to evaluate the relationship between the histomorphometric measurements and the duration of disease, age, or BAL cytology differential counts, as well as the correlation between the histomorphometric measurements within the same biopsy.

Descriptive statistics (median; range [min‐max]) were calculated for the semiquantitative score and histomorphometric measurements of the MMA and control horses.

Statistical analysis was performed with Prism software (GraphPad Software Inc. [version 7.0b], La Jolla, California). Differences were considered significant for *P* value ≤ .05.

## 3 RESULTS

Twelve horses with MMA, 6 mares and 6 geldings aged (mean ± SD) 7.1 ± 3.3 years (range, 2‐11 years) met the inclusion criteria. Eight horses were living in a stable and 4 in a pasture/paddock. All horses were fed with hay, had a history of exercise intolerance and 9 horses had a history of coughing episodes. Clinical signs first appeared when horses were aged 5.4 ± 3.1 years (range, 1‐9 years) and horses presented for signs lasting 1.6 ± 1.6 years (range, 1.5 months‐5 years). Thirteen horses were excluded on the basis of a history compatible with SA (9) and BAL fluid cytology findings (normal [3], and severe neutrophilia [41%; 1]). An additional 9 horses were excluded because there was no suitable biopsy available.

Eight control horses (1 research/teaching horse and 7 client‐owned) aged 10 ± 4.8 years (range, 5‐20 years), 4 mares and 4 geldings were included. Three horses were living in a stable, 4 in pasture/paddock and for 1 horse, the living conditions were not reported. They were all fed hay. Sixteen horses were excluded because of abnormal BAL fluid cytologic findings (neutrophilic inflammation [4], mast cell inflammation [2]), known concomitant diseases (9), and no biopsy available (1).

**FIGURE 1:**
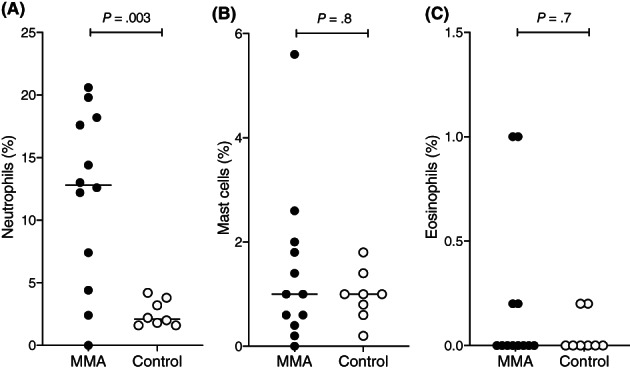
Bronchoalveolar lavage cytology differential counts. Neutrophils (A), mast cells (B), and eosinophils (C) in horses with MMA (n = 12) and control horses (n = 8). Horizontal lines represent the median. MMA, mild or moderate asthma

Seven horses with MMA had increased proportion of neutrophils in the retrieved BAL fluid, 3 horses increased mast cells and 2 horses had mixed inflammation (simultaneous neutrophilic and eosinophilic). However, only BAL fluid neutrophils were significantly increased in horses with MMA (*P* = .003; Figure 1) when compared with controls.

### 3.1 Histomorphometry

**FIGURE 2:**
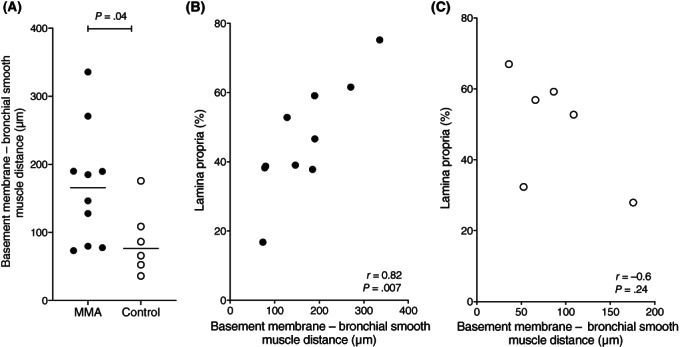
Distance between the basement membrane and the bronchial smooth muscle layer in MMA (n = 10) and control (n = 6) horses (A), correlation between the percentage of extracellular matrix in the biopsy (%) and distance between the basement membrane and the bronchial smooth muscle (μm) in horses with MMA (n = 10; black dots; B) and control horses (n = 6, white dots; C). Two horses from each group were excluded for this measurement because of the low quantity of smooth muscle in the biopsy. Horizontal lines represent the median. MMA, mild or moderate asthma

The thickness of the lamina propria, evaluated as the distance between the basement membrane and ASM, was significantly increased in horses with MMA (166 μm [73‐336 μm]) when compared with control horses (76 μm [36‐176 μm]; *P* = .04; Figure 2A). In horses with MMA (*r* = 0.81; *P* = .007; Figure 2B), but not in control horses (*r* = −0.60; *P* = .24; Figure 2C), this distance correlated with the relative area of the lamina propria. Ten horses with MMA and 6 control horses were included for these evaluations, as 2 horses from each group were excluded because of the low quantity of smooth muscle in the biopsy precluded performing the measurement.

**FIGURE 3:**
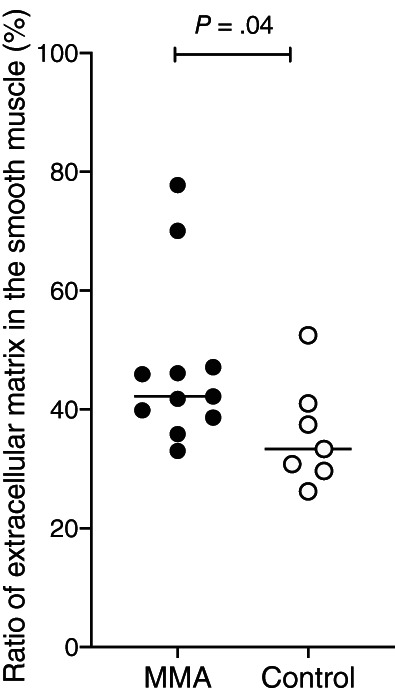
Smooth muscle fibrosis in horses with MMA (n = 11) and control horses (n = 7). One horse from each group was excluded for this measurement because of the small quantity of smooth muscle in the biopsy. Horizontal lines represent the median. MMA, mild or moderate asthma

The ratio of extracellular matrix in the smooth muscle evaluating smooth muscle fibrosis was significantly increased in horses with MMA (42% [33%‐78%]) when compared with control horses (33% [26%‐52%]; *P* = .04; Figure 3).

**FIGURE 4:**
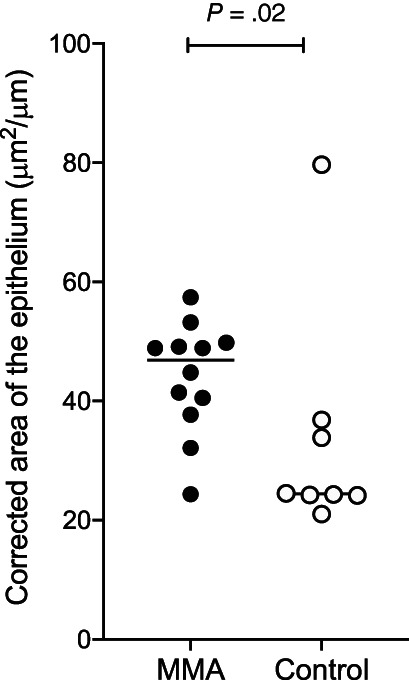
Epithelial hyperplasia in horses with MMA (n = 12) and control horses (n = 8). Horizontal lines represent the median. MMA, mild or moderate asthma

The epithelial hyperplasia evaluated by the corrected area of the epithelium was significantly increased in horses with MMA (47 μm^2^/μm [34‐57 μm^2^/μm]) when compared with control horses (24 μm^2^/μm [21‐80 μm^2^/μm]; *P* = .02; Figure 4). There were no differences in other histomorphometric measurements. The duration of the clinical signs was not associated with the remodeling of the airways (results not presented).

### 3.2 Semiquantitative histologic score

**FIGURE 5:**
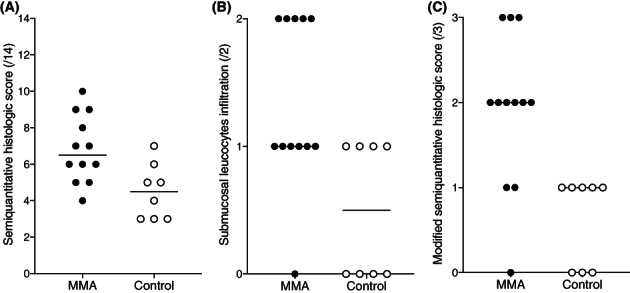
Semiquantitative histologic score (/14; A), submucosal leucocyte infiltration score (/2; B), and modified semiquantitative histologic score (/3; C) in horses with MMA and control horses. Horizontal lines represent the median. MMA, mild or moderate asthma

The semiquantitative histologic median score (/14) was 7 [4‐10] in MMA and 5 [3‐6] in control horses (Figure 5A).

The semiquantitative median score of epithelium in MMA and control horses were respectively 1 [0‐1] and 0 [0‐1] for epithelial hyperplasia (7 of the 12 horses with MMA and only 1 of the 8 control horses had a score of 1/1); 1 [0‐2] and 1 [0‐1] for inflammatory infiltrate; 0 [0‐0] and 0 [0‐1] for goblet cell hyperplasia; and 1 [0‐2] and 1 [0‐2] for epithelial desquamation. The semiquantitative median score of extracellular matrix in MMA and control horses were respectively 0 [0‐1] and 0 [0‐1] for the thickness of the basement membrane; 1 [0‐2] and 1 [0‐1] for submucosal inflammatory cells (Figure 5B); and 1 [0‐1] and 1 [0‐1] for mucous glands. Finally, the semiquantitative median score of ASM in MMA and control horses were respectively 1 [0‐2] and 1 [0‐2] for fibrosis; 0 [0‐1] and 0 [0‐0] for mucous glands; and 1 [0‐1] and 1 [0‐1] for smooth muscle ending visible.

Inflammation in the lamina propria scores and epithelial hyperplasia scores presented the higher difference between MMA and control medians compared to other criteria (median differences of 0.5 and 1, respectively). When these 2 criteria were combined, the score was 2/3 [0‐3] in MMA and 1/3 [0‐1] in control horses (Figure 5C).

## 4 DISCUSSION

This blinded and controlled study evaluated central airway remodeling in client‐owned horses with MMA. Results from this study revealed the presence of alterations of the central airway in horses with MMA as compared to nonasthmatic control horses. The airway epithelium, smooth muscle, and lamina propria were most affected, with epithelial hyperplasia, smooth muscle fibrosis, and thickened lamina propria being common changes. A rapid semiquantitative score also suggested remodeling in the epithelium (epithelial hyperplasia) and airway inflammation (leucocyte infiltration in the lamina propria) in MMA.

The thickening of the lamina propria present in horses with MMA occurs in horses with SA.^13^ As it is positively correlated with lung resistance in SA, these findings indicate this thickening of the lamina propria might also contribute to the airway obstruction in horses with MMA.^17^ The thickness of the lamina propria assessed by measuring the distance between the basement membrane and the smooth muscle layer was positively correlated with the extracellular matrix surface area, a measurement more difficult to obtain. Therefore, the thickness of the lamina propria as measured in the current study was appropriate to assess, and was faster to measure, than the surface area of the extracellular matrix. In control horses, these measurements were not correlated, possibly due to the small sample size.

The presence of epithelial hyperplasia was assessed by a single histomorphometric measurement and a semiquantitative histologic score. Correction of epithelial hyperplasia measurement was achieved by dividing the epithelial surface area by the basement membrane length. This allows for standardization of measurement of biopsies of different sizes and excludes mechanic desquamation, as only the well‐preserved epithelium areas were measured (rather than measurement of the entire epithelium surface area). The epithelium hyperplasia was increased in horses with MMA measured by histomorphometry. Some of the horses with MMA present no increased epithelial hyperplasia score (0/1), and a histomorphometric measurement similar to control horses. This finding could possibly be explained by the presence of 2 groups of horses with MMA related to the epithelial hyperplasia.

The semiquantitative histologic score (/14) developed to evaluate airway remodeling in the central airways of horses with SA^14^ revealed similarities in central airway remodeling in MMA and SA. Leucocyte infiltration in the lamina propria and epithelial hyperplasia were present in both conditions.^14^ A modified score, evaluating only these 2 parameters was rapidly performed under light microscopy, while the histomorphometric manual measurements were time consuming and require digitized images and the used of specialized software. However, other studies with a greater number of cases are needed to evaluate the usefulness of this score in MMA horses.

The smooth muscle surface area in central and peripheral airways is increased in horses with SA during exacerbation when compared to the remission state^21^, ^24^ and to control horses.^16^ Airway smooth muscle is central to airway hypersensitivity in humans, and this is likely to be true in horses.^32^ In the current study, however, the percentage of smooth muscle in the endobronchial biopsies was similar in both groups of horses. A comparable finding in horses with SA is reported.^13^ A proposed explanation is that the thickening of the lamina propria might have resulted in incomplete sampling of the smooth muscle layer.^13^, ^16^ This limits the value of endobronchial biopsies when assessing ASM via endobronchial biopsies. Evaluation of other indices of smooth muscle remodeling, such as myocytes density, cell proliferation, and apoptosis in endobronchial biopsies from horses with MMA would be feasible using these tissues, as they are found to be altered in horses with SA and do not require full thickness ASM sampling.^16^ A prospective study of horses with MMA using endobronchial ultrasound could also be considered as a noninvasive alternative to evaluating ASM remodeling, including progression of the disease over time, and after treatment.^12^ Bronchial smooth muscle fibrosis was significantly increased in horses with MMA compared to control horses. In horses with SA, the smooth muscle fibrosis is increased in peripheral and central airways.^24^

Although we started with a larger cohort, several horses did not meet inclusion criteria. A higher number of cases might have allowed more complete characterization of the remodeling features of MMA, and the presence of different remodeling phenotypes. Analyzing a higher number of biopsies by horses would have been preferable, but this was not possible in this client owned cohort. In a previous study,^24^ 5 biopsies are examined per horse, in contrast to 1 to 2 biopsies in the present study. Last, the exact carinas biopsied were not recorded. Nevertheless, the biopsies were collected from the most caudal to the most cranial carina until reaching the main carina, likely corresponding to 1.9 and 1.2 carina for the right lung and from 2.9 to 2.2 for the left lung.^13^

## CONFLICT OF INTEREST DECLARATION

Authors declare no conflict of interest.

## OFF‐LABEL ANTIMICROBIAL DECLARATION

Authors declare no off‐label use of antimicrobials.

## INSTITUTIONAL ANIMAL CARE AND USE COMMITTEE (IACUC) OR OTHER APPROVAL DECLARATION

Approved by the University of Montreal Animal Care Committee, number Rech‐1647.

## HUMAN ETHICS APPROVAL DECLARATION

Authors declare human ethics approval was not needed for this study.
